# In Vitro Morphogenesis of Tobacco: Modulation of Endogenous Growth Regulators by Tulsi (Holy Basil)

**DOI:** 10.3390/plants13142002

**Published:** 2024-07-22

**Authors:** Vanessa Vongnhay, Mukund R. Shukla, Murali-Mohan Ayyanath, Karthika Sriskantharajah, Praveen K. Saxena

**Affiliations:** Department of Plant Agriculture, Gosling Research Institute for Plant Preservation, University of Guelph, Guelph, ON N1G 2W1, Canada; vvongnha@uoguelph.ca (V.V.); mshukla@uoguelph.ca (M.R.S.); ayyanath@uoguelph.ca (M.-M.A.); sriskank@uoguelph.ca (K.S.)

**Keywords:** *Ocimum sanctum* L., shoot organogenesis, somatic embryogenesis, abscisic acid, zeatin, plant extract, regeneration

## Abstract

Plant growth regulators (PGRs) play a vital role in the induction of morphogenesis in vitro. Synthetic PGRs are commonly used to induce organogenesis and somatic embryogenesis from various explants, while natural substances are rarely utilized. This study aimed to enhance the regenerative response in *Nicotiana tabacum* leaf explants using Tulsi (*Ocimum sanctum*) leaf extract and to elucidate the biochemical interactions during modulation of endogenous plant growth regulators, including indole-3-acetic acid (IAA), abscisic acid (ABA), zeatin, and 6-(γ, γ-dimethylallylamino) purine (2iP). Tulsi leaf extract significantly improved shoot production through interactions between endogenous hormones and those present in the extract, which enhanced stress mitigation. The 20% Tulsi leaf extract treatment produced significantly more shoots than the control, coinciding with increased endogenous IAA and zeatin levels starting on day 10 in culture. Furthermore, ABA and zeatin concentrations increased on days 15 and 25, respectively, in the 20% Tulsi extract treatment, suggesting their role in the induction of somatic embryo-like structures. ABA likely acts as an activator of stress responses, encouraging the development of these structures. Additionally, 2iP was involved in the induction of both forms of regeneration in the 10% and 20% extract treatments, especially in combination with ABA. These results suggest that Tulsi leaf extract holds promising potential as a natural supplement for increasing plant regeneration in vitro and advancing our understanding of how natural extracts of plant origin can be harnessed to optimize plant regeneration processes in vitro.

## 1. Introduction

Plant cells exhibit totipotency, enabling them to form organs and entire plantlets, a developmental process regulated through the supplementation of various plant growth regulators (PGRs) [1]. These hormones induce and enhance regenerative processes within the explants, such as cell division, differentiation, and morphogenesis expressed as callus, shoot, root, and somatic embryo development [1]. There are several major classes of PGRs such as auxins, cytokinins, gibberellins (GA), abscisic acid (ABA), jasmonic acid (JA), and ethylene, most of which are also present endogenously [2]. Auxins and cytokinins are the most commonly added PGRs to the growth media. Auxins are involved in inducing the production of roots and callus, as well as cell elongation, while cytokinins stimulate cell division, lateral bud growth, and the formation of shoots [3]. Although PGRs within each of these classes act similarly, each PGR has unique effects depending on various factors, including species, concentration, environmental stressors, and combinations with additional growth regulators [4]. For instance, the natural auxin indole-3-acetic acid (IAA) is rapidly metabolized by plant tissues and is often less effective than the synthetic auxinic herbicide 2,4-dichlorophenoxyacetic acid (2,4-D), which is stable and most effective in inducing somatic embryogenesis due to its structural similarity to IAA [5].

Cytokinins play a vital role in signaling cell differentiation and the regeneration of shoots and somatic embryos. The natural cytokinin zeatin accumulates during the initial stages of shoot development, facilitating differentiation of the explant or callus into shoot buds and ultimately fully developed shoots [6,7,8]. The endogenous cytokinin 6-(γ, γ-dimethylallylamino)purine (2iP) also triggers the initiation of tissue directly into embryogenic callus [9,10] but is more widely distributed within the plant as a precursor for numerous other cytokinins [8]. Interactions between auxins and cytokinins are crucial, acting both synergistically and antagonistically to develop or inhibit the growth of callus, shoots, or roots depending on their ratio [11]. Research by Skoog and Miller [12] demonstrated that a high cytokinin-to-auxin ratio results in shoot production, while the opposite induces roots, and a balanced ratio produces callus.

Gibberellic acid (GA_3_) plays a key role in flowering and seed germination [13] but can act antagonistically to ABA in vitro, serving as a negative regulator of somatic embryogenesis in some species and a positive regulator in others [14]. ABA is involved in embryo maturation, synthesis of storage proteins, and defense against environmental stressors, often working in tandem with other hormones [15]. Increased levels of endogenous ABA in embryogenic callus are directly linked to stress treatments, resulting in somatic embryos [16]. JA also plays key roles in stress mitigation and defense responses, increasing in response to wounding and inducing signal cascades that lead to cytokinin accumulation at the wound site [17,18].

Several studies have investigated natural sources as supplements to the medium, such as coconut water, which is rich in natural cytokinins like trans-zeatin and trans-zeatin riboside, both effective in inducing callus and shoots [19,20]. Various plant hormones and growth regulators have also been identified in seaweed, tomato, banana, carrot, and papaya, with the potential to enhance in vitro growth and development [21,22]. Natural substances can often be superior to synthetic PGRs [23], providing a variety of organic nutrients, vitamins, sugars, amino acids, hormones, and stress resistance compounds and are often more affordable and attainable. Natural plant extracts reduce the reliance on synthetic chemicals, minimizing the risk of chemical contamination and potential side effects on the plant tissues. The complex mixture of phytohormones in plant extracts can also deliver a balanced and synergistic effect, promoting more efficient and effective morphogenesis compared to synthetic hormones [23,24]. Plant extracts contain secondary metabolites like flavonoids, phenolics, and alkaloids, which can influence regeneration by modulating growth pathways and protecting against microbial contamination. These compounds can act as signaling molecules, influencing cellular communication and differentiation during organogenesis. Recently, medicinal plant extracts have been explored to improve various stages of plant development [24]. For example, the application of various medicinal plant extracts showed a significant improvement in seed germination and seedling biomass accumulation in *Triticum aestivum*, likely due to the abundance of bioactive compounds mediating plant metabolism and activating the antioxidant defense system [24,25].

We recently investigated the role of *Ocimum sanctum* (Tulsi; also referred to as *O. tenuiflorum*) in mitigating browning and inducing regeneration through the modulation of indoleamine pathway [25]. This medicinal species is revered as the ‘Queen of Herbs’ due to its strong antioxidant and adaptogenic capabilities [26]. Further, Tulsi extracts can create optimized growth conditions that enhance the efficiency of organogenesis [25]. However, its interaction with various PGRs remains unknown. In this regard, we examined the effects of Tulsi leaf extract in varying concentrations on the interaction of various PGRs, including IAA, ABA, GA_3_, JA, BA, zeatin, and 2iP, which have been shown to play significant roles in in vitro regeneration. Thus, the objective of our research was to identify the influence of natural Tulsi leaf extract on regeneration pathways through the modulation of PGRs within the extract and the tobacco leaf explants grown in vitro.

## 2. Results

### 2.1. Effects of Tulsi Leaf Extracts on Organogenesis

*N. tabacum* leaf explants, which were cultured on media with increasing concentrations of Tulsi leaf extract, produced a mix of regenerants, which resembled shoots and embryo-like structures. The number of shoots was higher than that of the embryo-like structures (Table 1). All Tulsi leaf extract treatments (1%, 10%, and 20%) produced significantly (100%; *p* = 0.0622, <0.0001, 0.0143, respectively) more embryo-like structures than the control; however, the 10% concentration produced the greatest number somatic embryo-like structures per explant (2.50). Furthermore, the production of shoots was significantly (49%; *p* ≤ 0.0001) higher in the 20% Tulsi leaf extract treatment in comparison to the control group, which predominantly produced shoots. It is interesting to note that with the increase of Tulsi leaf extract concentration from 1% to 20%, the number of shoots also gradually increased; however, the difference between 10% and 20% leaf extracts was not significant.

### 2.2. Differences in the Mode of Regeneration of N. tabacum Explants

**Figure 1:**
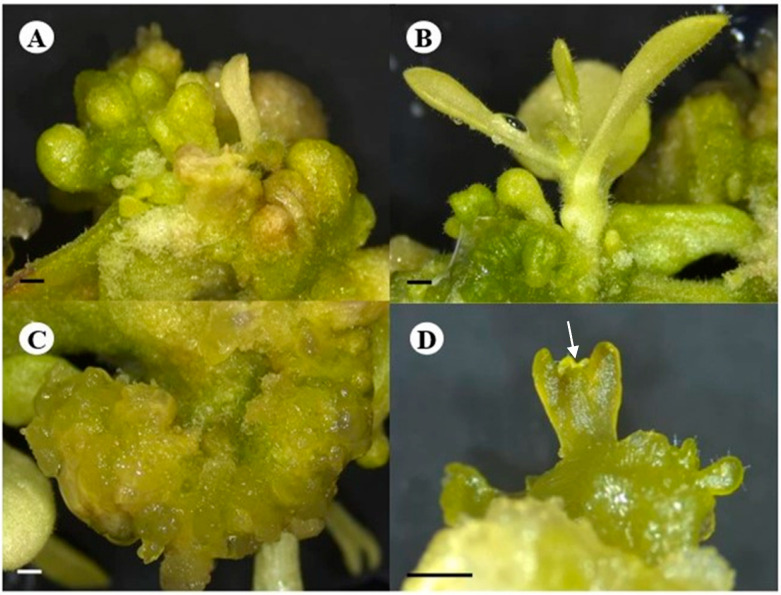
Different pathways of regeneration of the *N. tabacum* explants on the 20% Tulsi leaf extract treatment. (**A**,**B**) shoot development; (**C**) embryogenic callus; (**D**) cotyledonary stage with shoot apical meristem (white arrow). (**A**–**D**) bar = 1.0 mm.

The difference in the emergence of regenerants in cultures was also notable. The control treatment was first to develop shoot buds as browning occurred in the extract treated explants, yet the prevalence of shoot buds was visibly less when the control and the Tulsi leaf extract treatments were compared after 15 days of culture [25]. The shoot buds in control cultures developed into large shoots (approximately 2–3 cm in length), whereas the extract treatments had smaller but significantly more shoots, approximately 1 cm in length (Figure 1A,B). In addition to shoots, the control cultures also produced noticeably more friable callus throughout the culture period compared to the extract treatments, which produced callus with embryo-like structures around day 20.

After 20 days of culture, early stages of somatic embryo-like structures were visible within the embryogenic callus (Figure 1C), continuing to develop into the globular, heart, and cotyledonary shaped embryo-like structures (Figure 1D), which proceeded to form plantlets. The embryo-like structures were easily separated from the embryogenic callus for their continued development, as they were loosely attached to the explant. Furthermore, it is also worth noting that the development of the somatic embryo-like structures was asynchronous with different stages visible at different days in culture (Figure 1C,D).

### 2.3. Effect of the Extracts on PGRs

#### 2.3.1. Endogenous Levels of PGRs in Tulsi Leaf Extracts

**Figure 2:**
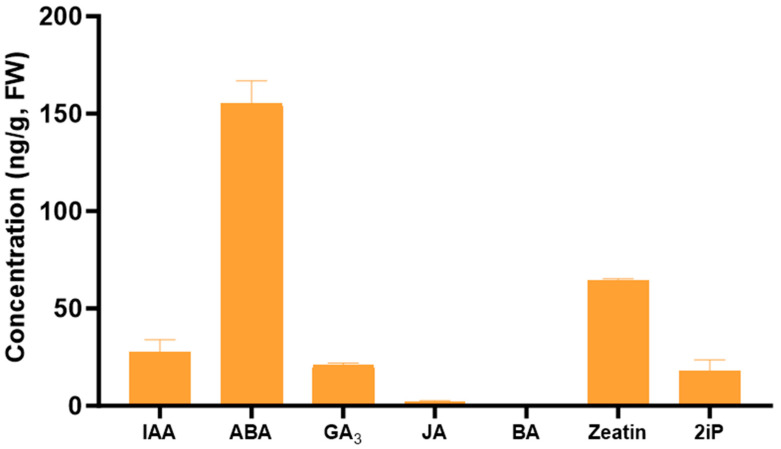
The concentration of the plant growth regulators indole-3-acetic acid (IAA), abscisic acid (ABA), gibberellic acid (GA_3_), jasmonic acid (JA), 6-benzylaminopurine (BA), zeatin, and 6-(γ, γ-dimethylallylamino)purine (2iP) in the Tulsi leaf extracts. Data represent the means ± SE of two biological replicates and three technical replicates.

Among the plant growth regulators quantified in the pure extract, all hormones, with the exception of BA, were detected in the Tulsi leaf extract (Figure 2). Of these, ABA was present in the greatest levels (155 ng/g FW), as well as zeatin present at 64 ng/g FW.

#### 2.3.2. Interplay among PGRs

##### Indole-3-Acetic Acid (IAA)

**Figure 3:**
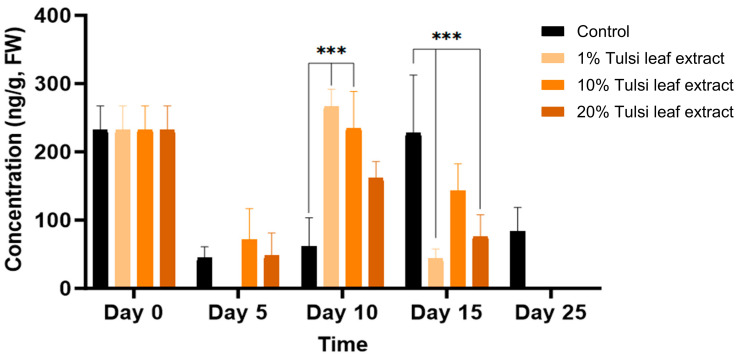
The concentration of indole-3-acetic acid (IAA) in the in vitro cultures of *N. tabacum* explants grown on media supplemented with various concentrations of Tulsi leaf extract over 0, 5, 10, 15 and 25 days. Data represent the means ± SE of two biological replicates and three technical replicates of each treatment and time point. *** Indicates significant differences at *p* < 0.0001 between the control and Tulsi leaf extract treatment based on Tukey’s HSD at α = 0.05.

The concentration of IAA throughout day 0 to day 25 noticeably fluctuated for all treatments. On day 0, IAA was detected in higher amount than day 5 (232 ng/g FW), at which time point IAA was not present in detectable quantities in the 1% Tulsi leaf extract treatment (Figure 3). On day 10, the 1% and 10% extract treatments showed significantly more IAA than the control (266 ng/g FW, *p* ≤ 0.0001; 234 ng/g FW, *p* ≤ 0.0001, respectively). However, after day 10, the IAA content in all Tulsi leaf extract treatments continuously decreased until non-detectable quantities were observed by day 25, while the control had significantly higher levels on day 15 (227 ng/g FW; *p* ≤ 0.0001).

##### Abscisic Acid (ABA)

**Figure 4:**
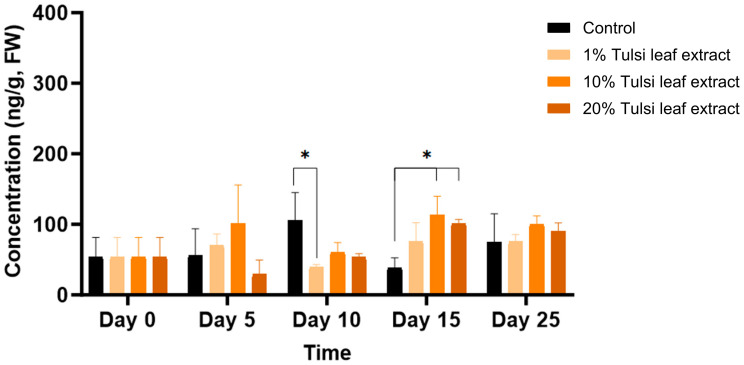
The concentration of abscisic acid (ABA) in the in vitro cultures of *N. tabacum* explants grown on media supplemented with various concentrations of Tulsi leaf extract over various days. Data represent the means ± SE of two biological replicates and three technical replicates of each treatment and time point. * Indicates significant differences at the *p* < 0.05 between the control and Tulsi leaf extract treatment based on Tukey’s HSD at α = 0.05.

ABA was detected in minimal quantities (54 ng/g FW) in the explants prior to culture and remained relatively constant until day 10, at which point the ABA content increased significantly compared to that in the 1% leaf extract treatment (106 ng/g FW, *p* = 0.0203). On day 15, ABA in the 10% and 20% leaf extract was present in significantly greater quantities (113 ng/g FW, *p* = 0.0044; 101 ng/g FW, *p* = 0.0403, respectively) than the control, although there was no significant difference among the treatments on day 25 (Figure 4).

##### Gibberellic Acid (GA_3_)

**Figure 5:**
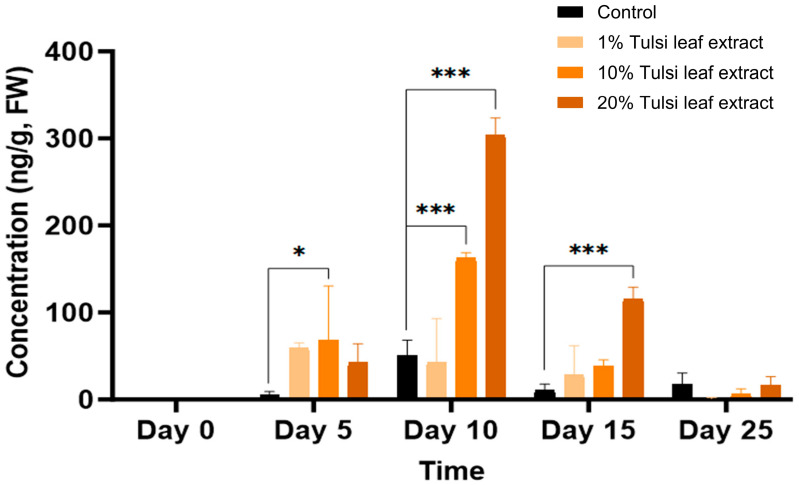
The concentration of gibberellic acid (GA_3_) in the in vitro cultures of *N. tabacum* explants grown on media supplemented with various concentrations of Tulsi leaf extract over 0, 5, 10, 15 and 25 days. Data represent the means ± SE of two biological replicates and three technical replicates of each treatment and time point. * Indicates significant differences at the *p* < 0.05 and *** at *p* < 0.0001 between the control and Tulsi leaf extract treatment based on Tukey’s HSD at α = 0.05.

Significant differences in GA_3_ content between the control and at least one of the extract treatments were observed across all time points, with the exception of day 25 (Figure 5). On day 5, the 10% Tulsi leaf extract treatment (68 ng/g FW, *p* = 0.0101) had significantly greater concentration of ABA than the control, which showed minimal quantities (5 ng/g FW). In addition to the significant increase in the 10% extract treatment (163 ng/g FW, *p* ≤ 0.0001) compared to the control, the 20% extract (304 ng/g FW, *p* ≤ 0.0001) on day 10 contained the highest concentration of gibberellic acid observed on any of the days in culture. The levels of GA_3_ were higher in all treatments compared to the control until day 15 (116 ng/g FW, *p* ≤ 0.0001) after which it was detected in minimal quantities on day 25.

##### 6-Benzylaminopurine (BA)

**Figure 6:**
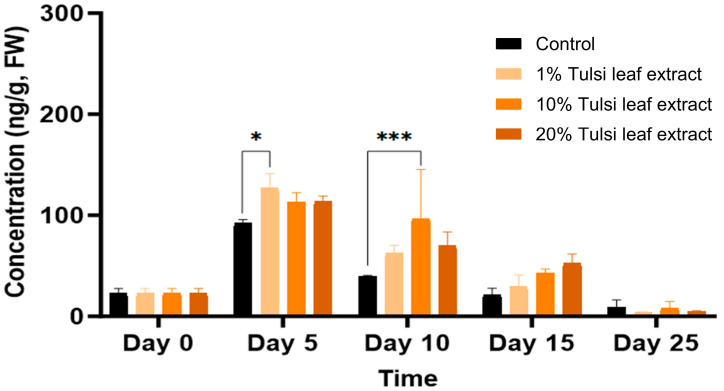
The concentration of 6-benzylaminopurine (BA) acid in the in vitro cultures of *N. tabacum* explants grown on media supplemented with various concentrations of Tulsi leaf extract over 0, 5, 10, 15 and 25 days. Data represent the means ± SE of two biological replicates and three technical replicates of each treatment and time point. * Indicates significant differences at the *p* < 0.05 and *** at *p* < 0.0001 between the control and Tulsi leaf extract treatment based on Tukey’s HSD at α = 0.05.

BA, which was supplemented to the culture medium in all treatments, was only present endogenously at 23 ng/g FW on day 0 but the concentrations thereafter increased significantly over day 5 to 15 (Figure 6). The explants on the media with 1% Tulsi leaf extract had significantly greater quantities than the control (127 ng/g FW; *p* = 0.0346). However, as the days in culture progressed and the supplemented BA was utilized by the explants, the concentration of BA continuously decreased, except for the 10% extract on day 10 (97 ng/g FW; *p* ≤ 0.0001).

##### Zeatin

**Figure 7:**
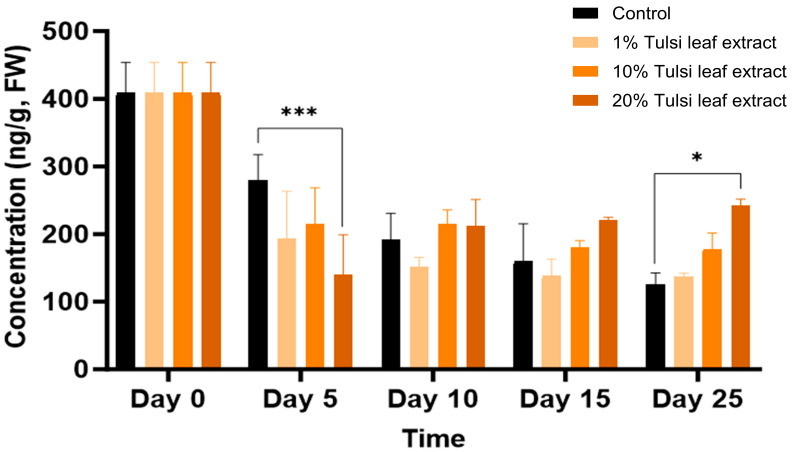
The concentration of the plant growth regulator zeatin in the in vitro culture of *N. tabacum* leaf explants grown on media supplemented with various concentrations of the Tulsi leaf extract over 0, 5, 10, 15 and 25 days of culture. Data represent the means ± SE of two biological replicates and three technical replicates of each treatment and time point. * Indicates significant differences at the *p* < 0.05 and *** at *p* < 0.0001 between the control and Tulsi leaf extract treatment based on Tukey’s HSD at α = 0.05.

Overall, zeatin was detected in all treatments over the duration of 0, 5, 10, 15, and 25 days in culture. Zeatin was detected at its highest concentration, approximately 400 ng/g FW on day 0, prior to the explants being cultured in vitro. Subsequently the concentrations showed a decreasing trend in all treatments on day 5 (Figure 7). Control on day 5 (280 ng/g FW) maintained the greatest concentration of zeatin compared to the Tulsi leaf extract treatments as seen with the 20% extract treatment (140 ng/g FW; *p* ≤ 0.0001). In treatments with both 10% and 20% extracts, no significant changes in the concentration of zeatin occurred for day 10 and 15, whereas on day 25 both leaf extract treatments showed significantly more zeatin (242 ng/g FW, *p* = 0.0015) than that detected in the control.

##### 6-(γ, γ-Dimethylallylamino)Purine (2iP)

**Figure 8:**
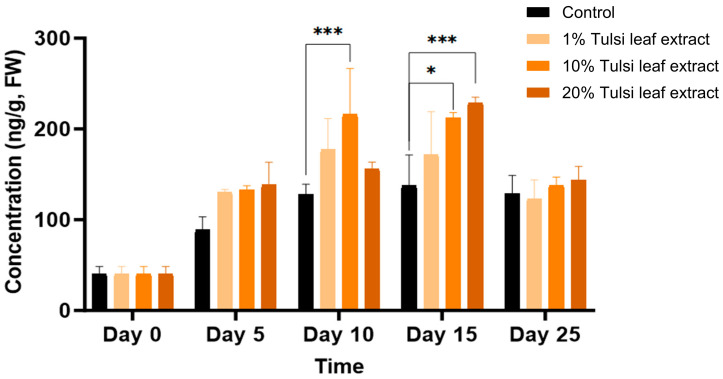
The concentration of the plant growth regulator 6-(γ, γ-dimethylallylamino)purine (2iP) in the in vitro culture of *N. tabacum* leaf explants grown on media supplemented with various concentrations of the Tulsi leaf extract over 0, 5, 10, 15 and 25 days. Data represent the means ± SE of two biological replicates and three technical replicates of each treatment and time point. * Indicates significant differences at the *p* < 0.05 and *** at *p* < 0.0001 between the control and Tulsi leaf extract treatment based on Tukey’s HSD at α = 0.05.

The natural cytokinin 2iP was observed to increase in quantities throughout day 0 to day 15, present at 41 ng/g FW on day 0 and significantly increasing on day 10 in the 10% Tulsi leaf extract treatment (216 ng/g FW; *p* ≤ 0.0001). A significant increase was observed on day 15, in both the 10% and 20% extract treatments (212 ng/g FW, *p* = 0.0012; 229 ng/g FW, *p* ≤ 0.0001), but not in the control. However, on day 25 all treatments showed decreased levels of 2iP with no significant differences among the concentrations (Figure 8).

##### Jasmonic Acid (JA)

**Figure 9:**
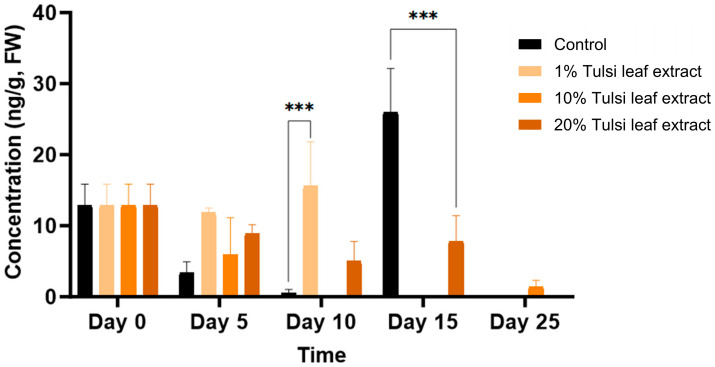
The concentration of jasmonic acid (JA) in the in vitro cultures of *N. tabacum* explants grown on media supplemented with various concentrations of Tulsi leaf extract over 0, 5, 10, 15 and 25 days. Data represent the means ± SE of two biological replicates and three technical replicates of each treatment and time point. *** Indicates significant differences at *p* < 0.0001 between the control and Tulsi leaf extract treatment based on Tukey’s HSD at α = 0.05.

Jasmonic acid was present at the lowest concentration of all the hormones tested, starting on day 0 with 13 ng/g FW. The JA concentration increased on day 10, at which time point the 1% Tulsi leaf extract treatment (16 ng/g FW; *p* ≤ 0.0001) had a significantly greater level than the control (0.6 ng/g FW) (Figure 9). Jasmonic acid was observed at its highest concentration on day 15 in the control, reaching 26 ng/g FW (*p* ≤ 0.0001), significantly greater than the 20% extract treatment. However, on day 25, with the exception of the 10% Tulsi leaf extract treatment, all treatments contained non-detectable quantities of the hormone.

#### 2.3.3. Interaction between Zeatin and Abscisic Acid in Tobacco Leaf Explants

**Figure 10:**
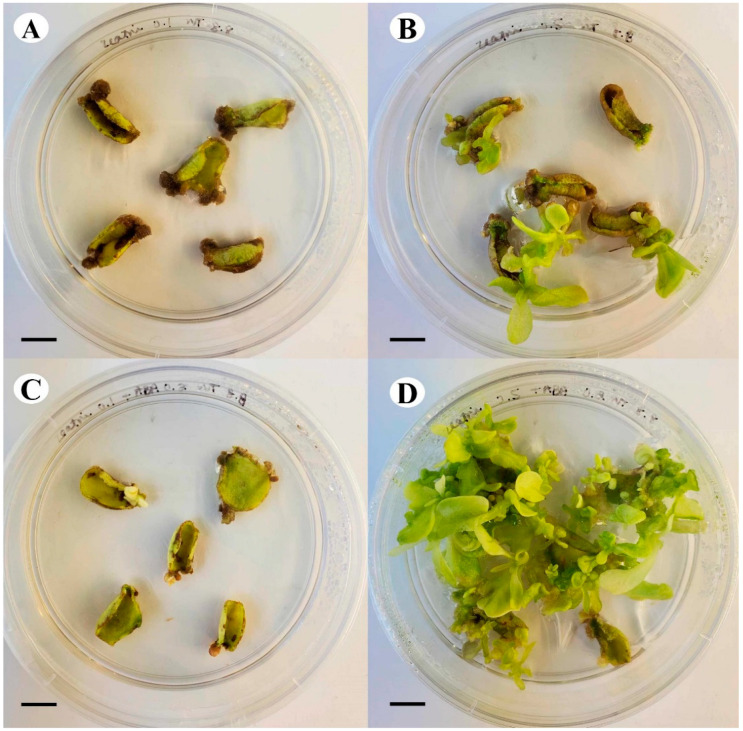
Comparison of growth and shoot development of tobacco leaf explants after 30 days in culture on (**A**) 0.1 μM zeatin, (**B**) 0.5 μM zeatin, (**C**) 0.1 μM zeatin + 0.2 μM ABA, and (**D**) 0.5 μM zeatin + 0.2 μM ABA (bar = 1.0 cm).

Tobacco leaf explants were cultured on various combinations of zeatin and ABA to investigate the influence of their interactions on the development of shoots. Over the course of 30 days in culture, the higher concentration of zeatin (0.5 μM) resulted in the development of more shoots than those observed with 0.1 μM zeatin, even when combined with ABA. The combination of 0.5 μM zeatin and 0.2 μM ABA induced the greatest number of healthy shoots, with the explants maintaining their green colour, compared to all other treatments (Figure 10), displaying the positive effect of zeatin and ABA interaction on shoot development.

## 3. Discussion

In this study, we investigated the potential of the extract of Tulsi, a widely used medicinal plant, in influencing the in vitro regeneration of tobacco leaf explants by modulating endogenous plant growth regulators. Our data demonstrated the significance of supplementing the medium with Tulsi extracts, which increased the regeneration of shoots in a concentration-dependent manner and enhanced the formation of somatic embryo-like structures. Additionally, the variation in the mode of regeneration might be attributed to the different hormone contents in the extracts. Tulsi leaf extract contained IAA, ABA, GA_3_, zeatin, and 2iP, while JA and BA were present in minimal quantities or were non-detectable. Cytokinins, whose sole addition results in shoot formation by promotion of cell division, are naturally present as trans-zeatin, cis-zeatin, and 2iP. These compounds have been shown to be effective in stimulating tobacco cell division, callus formation, and shoot development [20]. Another important endogenous hormone, ABA (abscisic acid), has gained interest due to its numerous roles, particularly its ability to improve desiccation tolerance and accelerate the maturation of somatic embryos. Generally present in minimal quantities until stressors occur, ABA acts as a key regulator of various physiological responses, similar to gibberellic acid, another crucial hormone examined in this research [15].

The natural auxin, IAA, was implicated in our study for its role in inducing the production of both types of regenerants: shoots and somatic embryo-like structures. IAA, which is crucial for key developmental processes such as embryogenesis, vasculature development, and apical dominance [27,28], was observed to significantly increase on day 10 in both the 1% and 10% Tulsi leaf extract treatments (Figure 3). This timing coincides directly with the initiation of shoots, similar to the observations by Cassels et al. [28], suggesting that the response of tobacco petiole explants was influenced by endogenous IAA levels. High IAA levels are linked to an increased potential for the production of shoots when used in conjunction with the cytokinin zeatin; both of these PGRs have been shown to localize to meristematic centers of shoot buds, indicating their signaling role in shoot differentiation [29,30]. Furthermore, the significantly higher concentration of IAA in the 10% Tulsi leaf extract, along with other bioactive compounds present, may be involved in the increased cell division associated with somatic embryogenesis. This treatment resulted in the greatest production of somatic embryo-like structures compared to all other treatments (Table 1). This phenomenon was also observed in *Cinnamomum camphora*, where the IAA/zeatin ratio was significantly higher in somatic embryos than other auxin/cytokinin combinations [31]. However, the role of IAA in embryogenesis significantly varies among species. For instance, in *Cunninghamia lanceolata*, reduced endogenous IAA resulted in increased production of somatic embryos, with IAA observed in its highest concentration in non-embryogenic callus [32]. Therefore, further research is necessary to understand the role of IAA in inducing embryogenesis across different plants when using medicinal plant extracts for stimulating morphogenesis.

ABA, which plays a crucial role in the synthesis of storage proteins in developing embryos and their maturation [33], was identified as a key hormone involved in the formation of somatic embryo-like structures in this study. All extract treatments significantly increased ABA levels compared to the control on day 15, coinciding with the differentiation of cells into embryo-like structures (Figure 4). This increase after approximately 10 days in culture mirrors the findings of Kamada and Harada [34], who observed a significant rise in endogenous ABA levels in carrot cells throughout the heart and torpedo stages of development. ABA likely served as an activator of stress responses within the plant in reaction to the numerous phenolic compounds present in the extracts, encouraging the development of somatic embryo-like structures [35]. Similar to how 2,4-D functions as a stress substance to induce the formation of somatic embryos, ABA has also been implicated in stress-induced embryogenesis [16]. This relationship has been reported in numerous studies, as embryogenic cell proteins, which are involved in embryogenic competence, are positively regulated by ABA [36,37]. Furthermore, in carrot, the concentration of endogenous ABA was significantly higher in embryogenic callus than in non-embryogenic callus in response to abiotic stress, highlighting the vital role of ABA in the induction of somatic embryogenesis [16,36]. Moreover, the level of both endogenous or exogenously applied ABA required to induce such regeneration is significantly lower than the amount of various auxins, such as IAA, which requires a 103-fold greater concentration than its endogenous level to be effective [38]. This suggests that the use of ABA is more effective in both mitigating stress within in vitro cultures and inducing the formation and maturation of somatic embryos [37,39].

In addition to ABA, GA_3_ was found to be significantly higher in the extract treatments compared to the control. On day 10, GA_3_ levels were at their highest in the 20% Tulsi leaf extract treatments, corresponding to the observed emergence of shoot buds (Figure 5). Typically, ABA and GA_3_ interact antagonistically, with ABA being a positive regulator and GA_3_ a negative regulator of somatic embryogenesis [40]. This antagonistic interaction likely occurred in the Tulsi leaf extract treatments, which produced high levels of GA_3_ consistently from day 5 to 15, resulting in fewer somatic embryo-like structures and increased shoots. It is possible that for this species and explant type, high ABA and low GA_3_ levels are required for the induction of embryogenesis, contrasting with the low ABA ratio observed in *Medicago truncatula* [14]. The consistent high levels of GA_3_ in the Tulsi leaf extract treatments may have shifted the balance towards organogenesis rather than embryogenesis, highlighting the species-specific and concentration-dependent nature of hormonal regulation in plant tissue culture.

BA is a commonly used cytokinin for the induction of in vitro regeneration process. In our studies, this synthetic hormone was supplemented to all treatments in addition to the auxin NAA. The increase of BA in all treatments on day 5, followed by a subsequent decrease over time, is logical as the supplemented BA in the medium was being utilized by the explants (Figure 6). However, the significant increase in BA in the 1% and 10% Tulsi leaf extract treatments on days 5 and 10, respectively, may be linked to BA’s role in shoot bud differentiation [41]. This observation aligns with findings by Bhanupriya and Kar [42], where the combination of BA with natural coconut water significantly enhanced the shoot induction response of the callus, ultimately leading to shoot formation. Additionally, various studies utilizing *N. tabacum* have shown that the highest frequency of shoots was observed with the addition of BA alone [43,44]. The interaction between BA and IAA may also be crucial in differentiating the various regeneration types. A high BA/low IAA ratio was observed to induce shoot organogenesis on day 5, while a low BA/high IAA ratio may be linked to the induction of somatic embryo-like structures on days 10 and 15 [45] (Figure 3 and Figure 6). This interplay highlights the importance of balancing these hormones to achieve desired regeneration outcomes.

The production of shoots and embryo-like structures can also be attributed to the presence of zeatin, both endogenously and supplemented through the extract. Typically, BA is used in place of natural cytokinins like 2iP and zeatin because it is more effective, inexpensive, and stable in vitro [46,47]. However, our results show that the addition of BA and NAA only induced the formation of fewer shoots in the control (Table 1), indicating that zeatin is a more effective PGR in our regeneration system. Zeatin was observed at its greatest concentration in the explants prior to culture. As a natural cytokinin, it is significantly higher in fully mature plants and shoots than in leaf explants, which is reflected by the decrease in zeatin levels thereafter (Figure 7). Interestingly, the level of zeatin detected in the control explants on day 5 was elevated compared to all Tulsi leaf extract treatments, coinciding with the earlier development of shoot buds in the control (Figure 1). This observation aligns with findings by Goebel-Tourand et al. [48], who noted that the addition of zeatin to the culture medium, especially in combination with indole-3-acetic acid (IAA), significantly reduced the time to shoot initiation compared to other cytokinin and auxin combinations. In contrast, the interaction between zeatin and NAA showed that exogenously applied NAA reduced the level of zeatin present, ultimately downregulating cytokinin signaling genes [49]. Furthermore, the concentration of zeatin in the 20% Tulsi leaf extract slowly increased beginning on day 10 and maintained a higher level of the cytokinin, while all other treatments remained at a lower level or decreased in the control. Zeatin has been observed to produce significantly more shoots in various species than other cytokinins [50,51]. Therefore, it is clear that zeatin played a key role in the production of numerous shoots, particularly in the 20% extract treatment, which produced significantly more shoots than the control.

Another natural cytokinin present in plants is 2iP. While both 2iP and zeatin are commonly used in in vitro cultures to promote axillary shoot production, zeatin has been observed in numerous studies to be both less phytotoxic and more effective than 2iP [52,53,54]. However, in our study, the significant increase of 2iP in both 10% and 20% Tulsi leaf extract treatments on days 10 and 15, respectively, may indicate a key role for this hormone in the induction of both organogenesis and somatic embryogenesis (Figure 8). It has been observed that the addition of 2iP to the culture media for *Coffea* species resulted in the induction of pro-embryogenic masses and fully developed embryos as the concentration of 2iP increased [55,56,57,58]. This cytokinin has been suggested to be more effective than BA, its synthetic counterpart, by enhancing cell division in pre-embryogenically determined cells [59]. Although 2iP may not be as effective as zeatin in inducing shoots, several studies have observed its effectiveness in various *Nicotiana paniculata* accessions, where its addition resulted in greater shoot production than that with other cytokinins [60]. This suggests its key role in both somatic embryogenic and organogenic regeneration. Furthermore, 2iP has been observed to be effective in combination with ABA. Supplementing with both 2iP and ABA induced green nodules in the callus as well as somatic embryos in date palm [61,62]. This combination underscores the synergistic potential of 2iP in enhancing regeneration processes when paired with other growth regulators.

The hormone JA (jasmonic acid) plays critical roles in the plant’s defense system and wounding related signaling. When damage or wounding occurs to any part of the plant, wound signals are produced, triggering a signal cascade that elicits calcium ion signals, the production of reactive oxygen species (ROS), and increased levels of hormones like JA and SA (salicylic acid) [17,63]. These hormones are typically present in minimal quantities and only spike in response to wounding signals [18]. This pattern was observed in our study, where JA levels initially increased after the leaf discs were cut and then decreased across all treatments, with the exception of the 1% Tulsi leaf extract and the control on days 10 and 15, respectively (Figure 9). The wounding process causes the accumulation of cytokinins at the wound site, in this case, the edges of the leaf disc, and promotes the upregulation of cell-cycle-related genes. This, in turn, induces wound-induced callus formation, which can be directed into organogenic or somatic embryogenic regeneration by additional endogenous hormones [18,64]. The significant increase of JA in the 1% Tulsi leaf extract treatment may be linked to the induction of shoot organogenesis. This was similarly observed by Park et al. [65], who found that treating *Arabidopsis* explants with JA induced shoot organogenesis. The prominence of JA in the control, while the hormone was in minimal quantities in all other treatments, was likely due to the antioxidant properties of JA. The signaling cascade triggered by JA elicits the production of various secondary metabolites involved in ROS scavenging. In contrast, the Tulsi extracts contain a cocktail of compounds with antioxidant properties [25], so these extract treatments did not require JA to perform the same functions. This suggests that the Tulsi extracts provide sufficient antioxidant activity, thereby reducing the need for JA in the treated explants.

The interaction between zeatin and ABA is also prominent, as healthy shoots primarily developed in the presence of ABA and a higher zeatin concentration (Figure 10). This preliminary experiment underscores the importance of both hormones and their interaction, an observation also noted by Ammirato [66], who found that a precise balance between ABA and zeatin is key in the production of various regenerants. In our study, the higher concentration of zeatin supplemented to the media likely resulted in the formation of shoots (Figure 10).

Overall, the addition of Tulsi leaf extracts to the media improved regeneration by supplementing and mediating the levels of several endogenous hormones. These natural extracts enhanced the balance and availability of critical hormones such as zeatin and ABA, thereby promoting the development of both shoots and somatic embryo-like structures. These observations highlight the potential of using natural plant extracts to improve in vitro culture conditions and regeneration outcomes.

## 4. Materials and Methods

### 4.1. Growth of Plant Materials and Preparation of Extracts

Tulsi germplasm line ‘Vrinda’ identified by Shukla et al. [67] was utilized for preparation of leaf extracts. Plants were grown and maintained at 23 °C and 45% relative humidity in the greenhouse (University of Guelph, Guelph, ON, Canada). Once the Tulsi plants reached full maturity and were flowering, healthy leaves were collected and dried at 55 °C in a drying room for three days. Extracts were prepared from the dried leaves using 1 L of heated (75 °C) double distilled water and stirring 10 g of powdered leaves for 30 min. After sterilizing the solution with a Welch vacuum (0.2 μm pore size; Ideal vacuum, Albuquerque, NM, USA), it was stored at room temperature.

### 4.2. Nodal Culture Establishment of Tobacco Explants

*N. tabacum* plant material was obtained and used following the protocol described earlier by Vongnhay et al. [21], in which the tobacco leaf discs were cultured on MS basal medium with 2.0 μM 6-benzylaminopurine (BA) + 0.2 μM 1-naphthaleneacetic acid (NAA) + 3% sucrose (control), added prior to autoclaving. The sterile Tulsi leaf extract (1%, 10% or 20%) was added after autoclaving while maintaining the total medium volume constant. The medium lacking Tulsi leaf extract was used as the control. These cultures were maintained at 25 °C under “cool white” fluorescent tubes at 20–25 μmol m^−2^ s^−1^ light intensity in a 16-hr photoperiod.

#### Tobacco Leaf Disc Explants Culture and Sample Collection

Thirty leaf explants (0.8 cm discs; 5 per plate) were used to test the treatment effects, and all experiments were repeated twice. All primary cultures were examined daily for the effects of the extracts on regeneration either in the form of organogenic shoots or somatic embryo-like structures. After 4 weeks in culture, both pathways of regeneration were investigated and compared between the control and all Tulsi leaf extract concentration treatments. Regeneration was quantified by recording organogenic shoots greater than 1 cm in size and somatic embryo-like structures at the globular or more developed stages per explant. For biochemical analysis, explants from each treatment were collected on days 0, 5, 10, 15 and 25. Media was removed, and the explants were placed in −80 °C until further analysis.

To further elucidate the role and interaction between zeatin and ABA, an experiment following the same growth conditions as above was conducted utilizing tobacco leaf explants cultured on MS basal media with 3% sucrose and supplemented with either (a) 0.1 μM zeatin, (b) 0.5 μM zeatin, (c) 0.1 μM zeatin + 0.2 μM ABA, or (d) 0.5 μM zeatin + 0.2 μM ABA. Observations of the growth and development of shoots were taken after 30 days of culture.

### 4.3. Detection and Quantification of Hormones

#### 4.3.1. Sample Collection and Extraction

All eight hormones were detected and quantified by Ultra Performance Liquid Chromatography—Mass Spectrometry (UPLC-MS). Samples were prepared following the methanol double extraction protocol described by Ayyanath et al. [68]. Leaf samples from each time point were finely powdered and extracted using 500 μL of extraction solvent, comprised of UPLC–MS grade 75% methanol and 5% formic acid (Fisher Chemical, Ottawa, ON, Canada) in Milli–Q water. After vortexing and storing the solution at −20 °C for 1 h, it was then spun down at 14,000 rpm at 4 °C for 15 min (Sorvall ST 8R, ThermoFisher Scientific, Dreieich, Hessen, Germany). The supernatant was collected and kept at −20 °C until the second extraction was completed. These steps were repeated to collect the solution from the second extraction and pooling the supernatants. Using the Solid Phase Extraction (SPE) technique described by Yalçın et al. [69], the pooled supernatant was purified and preconcentrated. The metabolites retained in the SPE cartridge (Oasis^®^ HLB, 1 cc, Waters, Beverley, MA, USA) were eluted with 200 μL methanol and were then filtered through a 0.22 μM centrifuge filter (Millipore; 1 min, 13,000 rpm). The flow-through was immediately analyzed on UPLC-MS.

#### 4.3.2. Separation and Quantification

To separate the hormones from the collected solution, reverse phase ultra-performance liquid chromatography system was utilized (UPLC: Shimadzu LabSolutions, Columbia, MD, USA). This was performed by injecting 5 μL aliquot of sample onto Acquity BEH column (2.1 × 50 mm, 1.7 μm; Waters, Beverley, MA, USA). The hormones were separated with a gradient of solvents A (0.1% formic acid, pH 3.0) and B (100% acetonitrile) with initial conditions at 95% A (5% B) increased to 5% A (95% B) over 5 min using an empower curve of 8. The column temperature was 40 °C, and the flow rate was 0.2 mL min^−1^.

The hormones zeatin, ABA, benzyl adenine (BA), and 6-(γ, γ-dimethylallylamino) purine (2iP), were detected in positive mode for mass-to-charge (*m*/*z*) of 220, 265, 226, and 204 while the hormones IAA, GA_3_, jasmonic acid (JA), and salicylic acid (SA) were detected in negative mode for mass-to-charge (*m*/*z*) of 176, 345 209 and 137, respectively by using a single quadrupole mass spectrometer (LCMS 2020, Shimadzu, Kyoto, Japan) in a single ion recording mode (SIR). The standards utilized were analytical grade and purchased from Sigma Aldrich (Oakville, ON, Canada). The instrument probe temperature was set to 250 °C with a gain of 5 and then capillary positive and negative voltages were set to 0.5 kV. To compare and quantify the eight hormones analyzed, standard curves were generated for each hormone in respect to their corresponding standards. Data from two biological and two technical replicates were used for the calculations (LabSolutions, v5.109/2020, Shimadzu Corporation, Kyoto, Japan).

### 4.4. Statistical Analysis

Data from the randomized experiment design were subjected to analysis of variance (ANOVA) using PROC GLIMMIX in SAS^®^ Studio (SAS Institute Inc., Cary, NC, USA). The data were tested for normalization using Shapiro-Wilk normality tests, and the error assumptions of variants were analyzed using homogenous and studentized residual tests. The significant differences between the control and 1%, 10% and 20% Tulsi leaf extract treatments at each time point were determined using Tukey’s Honest Significant Different (HSD) multiple comparison test (α = 0.05). Means ± SE per each PGR and organogenic and somatic embryogenic-like regeneration types were presented in graphical and tabular formats, respectively.

## 5. Conclusions

In conclusion, the findings of this study provide evidence of the positive influence of medicinal plant extracts in inducing increased regeneration in the form of organogenesis and somatic embryogenesis. Tulsi leaf extract significantly improved in vitro regeneration and mitigated stress through interactions between the endogenous hormones oS1f the explants and those present in the extract. Additionally, the significant production of somatic embryo-like structures was attributed to the roles of endogenous and supplemented ABA and IAA. Future research should investigate the effects of these medicinal extracts at a genetic level to determine if they cause permanent changes passed through generations of subcultures. This avenue of research holds tremendous promise for advancing our understanding of how natural extracts can be used to improve plant tissue culture techniques.

## Figures and Tables

**Table 1:** Comparison between control and Tulsi leaf extract on the induction of regenerants including shoots and somatic embryo-like structures in cultured *N. tabacum* leaf discs. Values represent means ± SE, and different letters within a column represent significant differences between the treatments based on Tukey’s HSD test at α = 0.05. The numbers of regenerants, shoots, and embryo-like structures represent average per explant from 30 explants.

Treatment	Average Number of Regenerants/Explant	Average Number of Shoots Greater than 1 cm/Explant	Average Number of Embryo-Like Structures
Control	6.14 ± 0.5 c	6.14 ± 0.5 c	0 c
1% Tulsi leaf extract	9.60 ± 0.5 b	8.75 ± 0.5 b	0.98 ± 0.5 cb
10% Tulsi leaf extract	12.58 ± 0.5 a	10.58 ± 0.5 a	2.50 ± 0.5 a
20% Tulsi leaf extract	13.07 ± 0.5 a	12.05 ± 0.5 a	1.53 ± 0.5 b

## Data Availability

Data are contained within the article and Supplementary Materials.

## Supplementary Materials

The following supporting information can be downloaded at: https://www.mdpi.com/article/10.3390/plants13142002/s1, Table S1: Adj *p* values of the plant growth regulators at various developmental stages using the Tulsi leaf extract. Figure S1: The fresh weight concentration of SA in the in vitro cultures of tobacco supplemented with Tulsi leaf extract on day 0, 5, 10, 15 and 25.
