# Supplementary material for: In Vitro Morphogenesis of Tobacco: Modulation of Endogenous Growth Regulators by Tulsi (Holy Basil)
Source: Plants (Basel). 2024 Jul 22;13(14):2002. doi: 10.3390/plants13142002 (PMC11280594; doi:10.3390/plants13142002)
Supplement: Supplementary file 1 [file plants-13-02002-s001.zip › plants-3114295-supplementary.pdf]

Table S1. Adj P-values of various control and Tulsi leaf extract treatments compared on day 5, 10, 15 and 25 in vitro.

Treatment_Day	Adj.P values (fresh weight concentration)						
	IAA	ABA	GA_3	JA	BA	Zeatin	2iP
**C_5 vs 1% Tulsi leaf extract_5**	0.9141	1.0000	0.0567	0.2438	0.0346	0.0621	0.4337
**C_5 vs 10% Tulsi leaf extract _5**	0.9999	0.5626	0.0101	0.9999	0.7544	0.4285	0.3484
**C_5 vs 20% Tulsi leaf extract _5**	1.0000	0.9923	0.5786	0.7252	0.6874	<.0001	0.1684
**C_10 vs 1% Tulsi leaf extract _10**	<.0001	0.0203	0.7891	<.0001	0.6181	0.9711	0.1691
**C_10 vs 10% Tulsi leaf extract _10**	<.0001	0.4132	<.0001	1.0000	<.0001	1.0000	<.0001
**C_10 vs 20% Tulsi leaf extract _10**	0.1296	0.1846	<.0001	0.9374	0.1400	1.0000	0.9440
**C_15 vs 1% Tulsi leaf extract _15**	<.0001	0.7503	0.5655	<.0001	1.0000	1.0000	0.7603
**C_15 vs 10% Tulsi leaf extract _15**	0.0564	0.0044	0.968	<.0001	0.7203	1.0000	0.0012
**C_15 vs 20% Tulsi leaf extract _15**	<.0001	0.0403	<.0001	<.0001	0.1162	0.562	<.0001
**C_25 vs 1% Tulsi leaf extract _25**	0.0658	1.0000	1.0000	1.0000	1.0000	1.0000	1.0000
**C_25 vs 10% Tulsi leaf extract _25**	0.0658	0.9912	1.0000	1.0000	1.0000	0.808	1.0000
**C_25 vs 20% Tulsi leaf extract _25**	0.0658	1.0000	1.0000	1.0000	1.0000	0.0015	1.0000

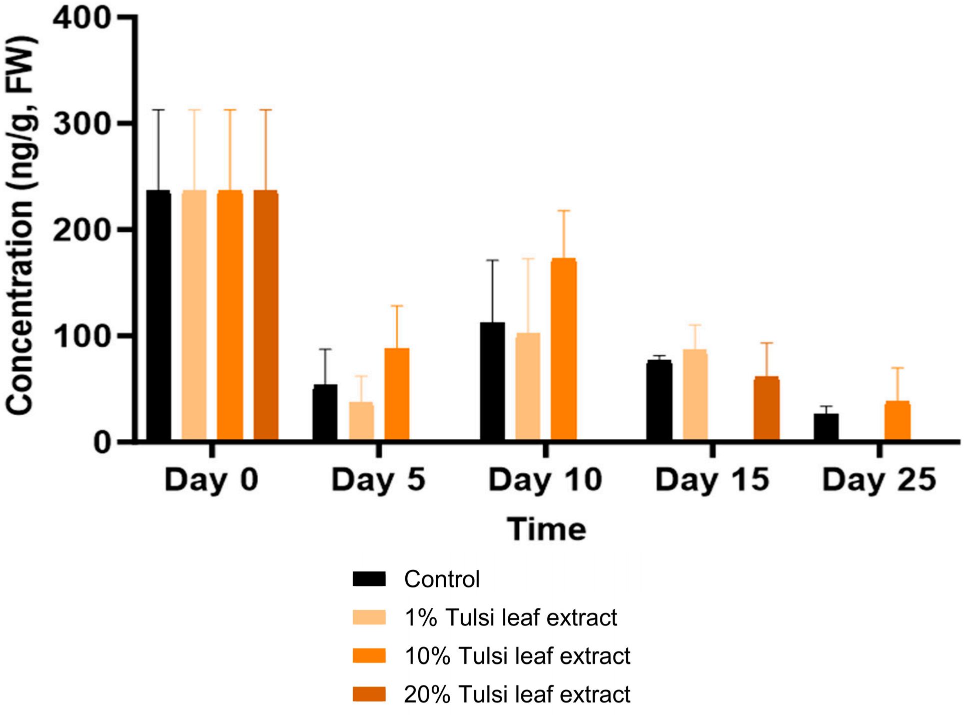

Figure S1. The fresh weight concentration of salicylic acid (SA) in the in vitro cultures of *N. tabacum* supplemented with various concentrations of Tulsi leaf extract over 0, 5, 10, 15,and 25 days in culture. Data represents the means ± SE of two biological replicates and three technical replicates of each treatment and time point.
